# Predicting Mechanical Responses in Polymer Blends with Unintended Polymer Fractions Using an Efficient Neural Network-Based Constitutive Material Model

**DOI:** 10.3390/polym17070963

**Published:** 2025-04-01

**Authors:** Ninghan Tang, Pei Hao, Juan Miguel Tiscar, Francisco A. Gilabert

**Affiliations:** 1 Department of Materials, Textiles and Chemical Engineering (MaTCh), Mechanics of Materials and Structures (MMS), Tech Lane Ghent Science Park-Campus A, Ghent University (UGent), Technologiepark-Zwijnaarde 46, 9052 Ghent, Belgium; ninghan.tang@ugent.be (N.T.); pei.hao@ugent.be (P.H.); 2 Instituto de Tecnología Cerámica (ITC), Asociación de Investigación de las Industrias Cerámicas (AICE), Universitat Jaume I, Campus Universitario Riu Sec, 12006 Castellón, Spain; juanmiguel.tiscar@itc.uji.es

**Keywords:** polymer blends, mechanical recycling, thermomechanical behavior, neural network, constitutive modeling, stress–strain predictions

## Abstract

Current mechanical recycling procedures often fall short of achieving 100% purity in recycled thermoplastics, which typically consist of mixed polymer types. These other polymers, though typically present in small amounts, can significantly affect the mechanical properties of the recycled material. Addressing this issue, this study introduces a neural network (NN) approach combined with a physically-based constitutive model to accurately predict the mechanical behavior of polymer blends of varying compositions. The NN-based method relies on the training of a crucial internal variable controlling the nonlinear response. This variable is derived from the physical model, which minimizes the dependence on extensive experimental data. We evaluated this approach on polymer blends of LLDPE/PET, LLDPE/PA6, and LDPE/PS at various weight fraction ratios. The results demonstrate that the NN-based model effectively aligns with experimental outcomes, enhancing our ability to predict how different blend ratios influence the mechanical properties of polymer blends. This capability is crucial for optimizing the use of recycled polymers in various applications.

## 1. Introduction

Plastics are widely used as single-use products due to their advantageous properties, including light weight, low cost, ease of processing, and excellent mechanical performance [1]. In 2023, global plastic production reached 413.8 Mt [2]. According to Eurostat [3], the European Union generated an average of 36.1 kg of plastic packaging waste per person in 2022. A significant portion of this waste consists of multilayer materials [4], which are composed of different types of polymers that are challenging to separate. This difficulty in separation often leads to cross-contamination during the recycling process.

Due to inherent challenges in separating and purifying polymers, recycled plastics often exist as polymer blends containing small fractions of other polymers. Recently, some studies have been conducted to investigate the mechanical behavior of polymer blends [5,6,7]. Experimental results indicate that polymer blends exhibit significantly different mechanical properties compared to pure polymers, which creates challenges in modeling and optimizing their mechanical performance [8]. Therefore, incorporating the effect of weight fraction is crucial for accurately modeling the mechanical behavior of polymer blends.

Several models have been proposed to explore the relationship between mechanical properties and polymer mixing ratios, aiming to achieve the desired mechanical performance for applications in the automotive industry, flexible electronics, biomedical devices, and packaging [9,10,11,12]. To comprehensively describe the mechanical response of polymer blends, Fang et al. [13] developed a phenomenological model specifically for polycarbonate and acrylonitrile–butadiene–styrene (PC/ABS) blends. By using digital image correlation (DIC) to observe the large deformation of PC/ABS blends, a six-parameter constitutive model was proposed that accounts for the strain rate effects. Hund et al. [14] conducted experiments to explore the large strain deformation and fracture behavior of rubber-toughened PC/ABS blends with two compositions. The suitability of three material models, the Drucker–Prager, the Raghava, and the Green/Gurson-like models, to predict the macroscopic stress–strain response and fracture behavior under uniaxial and more complex loading conditions is assessed through finite element simulations. the results show that the applicability of the PC/ABS material model also depends on the blend composition. Additionally, Hentati et al. [15] assessed the effectiveness of different existing models and developed a phenomenological constitutive model named the Hentati–Mnif–Hfaiedh–Petit (HMHP) model that refers to the Zhu et al. [16] model to better predict the thermomechanical behavior of PC/ABS blends at varying temperatures and strain rates.

However, polymer blends commonly found in recycled multilayer packaging plastics—such as polyethylene terephthalate (PET), polystyrene (PS), linear low-density polyethylene (LLDPE), and low-density polyethylene (LDPE)—are often less studied recently. Moreover, most existing models tend to focus on a single mixing ratio [15] or rely on separate parameter sets for each blend composition [17]. This limitation prevents a comprehensive understanding of the stress–strain response of blends and obscures the role of weight fraction in determining mechanical properties, such as effective elasticity, the onset and evolution of plasticity, and more specific details related to load rate effects.

The integration of machine learning (ML) approaches has demonstrated significant effectiveness in materials science, particularly in the fields of material modeling and multiscale analysis [18,19,20]. Through these advanced simulation techniques, the nonlinear mechanical response of a wide range of materials, including metals [21], composites [22], biological materials [23,24], and polymers [25], has been successfully investigated. More specifically, in the context of material modeling for polymer blends, Yousef et al. [26] developed a NN model to capture the mechanical response of polyethylene (PE), polypropylene (PP), and their blends. The model uses strain and blend ratios as inputs to predict stress, effectively capturing stress–strain curves even when the blend composition is unseen by the pre-trained NN. However, the approach relies on experimental data for NN training, considering strain and stress in the tensile test direction, which limits its implementation in finite element methods for 3D simulations. Additionally, the model treats the NN as a black-box approach, fitting the stress–strain relationship without incorporating constitutive models that would provide deeper insights into the mechanical response. Moreover, polymer blends’ typical mechanical features such as temperature, strain rate, and pressure dependence are not accounted for in this model. To investigate the relationship between the elastic modulus and volume fraction, Sharifzadeh and Amiri [27] created an NN-based model that accounts for morphological variations and incorporates critical series/parallel sections as well as polymer/polymer interfaces to predict the tensile modulus of binary polymer blends. Although this model is limited to 1D stress simulations, the study underscores the potential of artificial neural networks (ANNs) as a promising and efficient analytical tool for reducing both cost and time in polymer characterization.

**Figure 1:**
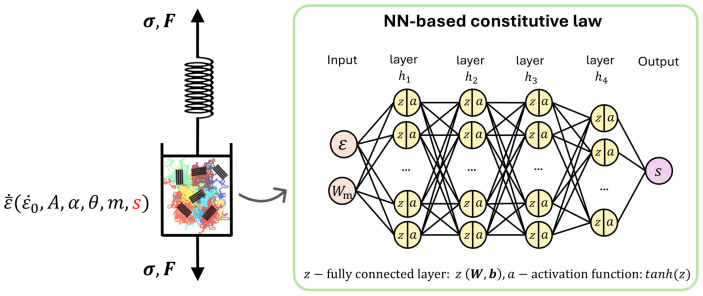
Schematic diagram of the FEM-NN-based constitutive model. The left side represents the intermolecular resistances in the USCP model, while the right side illustrates the structure of the NN. The inputs to the NN are ε and $W_{m}$, where ε represents the current strain during the loading process, and $W_{m}$ denotes the weight fraction of the main composition. The output of the NN is *s*, which corresponds to the athermal shear resistance.

In this study, we propose a constitutive model that integrates the finite element method (FEM) and an NN based on the theoretical foundations of polymer-blend modeling. The model is designed to investigate how variations in the ratio of two polymers influence key mechanical properties of polymer blends. Instead of directly establishing a stress–strain relationship using NNs, this method focuses on predicting the value of an internal factor, “*s*”, termed as athermal shear resistance, which is a single scalar magnitude that controls the kinematics of the viscoplastic evolution of the polymer. This magnitude is the core of the advanced physical-based model illustrated in Figure 1. By adopting this approach, we can significantly reduce the reliance on extensive experimental testing campaigns, simplify the parameter identification process, and enhance the model’s generalization capabilities. Additionally, it enables efficient and rapid exploration of various mechanical scenarios and material combinations. To validate the FEM-NN model, we consider three polymer-blend systems commonly derived from recycled multilayer packaging plastics: LLDPE/PET, LLDPE/PA6, and LDPE/PS. Our work not only addresses critical challenges in the utilization of recycled plastics but also provides valuable insights for designing high-performance polymer blends tailored to industrial applications.

The structure of this paper is organized as follows: Section 2 outlines the theoretical background of the constitutive model used to generate the synthetic data required for the weight fraction-dependent NN-based polymer model. This section also introduces a technique to mitigate the rate-dependent effects from the original constitutive model, enabling a more efficient isolation of the role of polymer blend composition. Section 3 details the calibration process and presents the simulation results for three polymer blends. Section 4 provides a comprehensive description of the NN-based constitutive model, including the training methodology. Section 5 discusses the validation results and presents a series of predictions derived from the FEM-NN model. Finally, Section 6 summarizes the key findings, concludes the paper, and proposes future research directions.

## 2. Constitutive Modeling

To develop the hybrid FEM-NN approach, this work employs a physics-based constitutive model known as the Unified Semi-Crystalline Polymer (USCP) model, proposed by Hao et al. [28]. The USCP model is grounded in the double-kink release theory [29] and effectively captures the rate-, temperature-, and crystallinity-dependent behavior of both thermoplastics and thermosets.

The identification of material coefficients in the USCP model requires experimental stress–strain curves obtained under at least two strain rates. This is essential for determining the rate-sensitivity parameters, which will be discussed in detail later. Although the USCP model is specifically designed to investigate the influence of loading rate on the mechanical behavior of polymers, this research introduces a slight reformulation to render the model rate-independent. The primary objective of this study is to assess the influence of weight fraction on the stress–strain response of various polymer blends. Therefore, existing experimental data often explore various mix ratios of polymer blends without considering the strain rate. Therefore, the limited experimental data are insufficient to calibrate the USCP model, which requires at least one stress–strain curve and a peak stress value at a different strain rate. To make this model more practical for industrial applications, the strain-rate dependence has been deliberately excluded by simplifying the USCP model, and the effect of the secondary polymer in the blend is isolated. The combined effect of strain rate will be addressed in future research.

In this section, to distinguish it from the original USCP model, the new rate-independent formulation is termed the USCP-Lite (USCP-L) model. The following sections will describe both models in detail and explain the strategy used to derive the Lite version.

### 2.1. The USCP Model in Brief

The USCP model was proposed with the aim of providing a comprehensive framework to accurately capture the thermomechanical behavior of semi-crystalline polymers (SCPs), particularly under conditions influenced by strain-rate-triggered self-heating effects. The model addresses the challenges of characterizing a wide variety of thermosets and thermoplastics, offering a unified approach for understanding their large deformation and temperature- and strain rate- dependence. Addtionally, the USCP model can also accurately capture the double-yield (DY) phenomenon of polymers [28,30].

A thermally activated viscoplastic flow was proposed by Argon [29] to account for the temperature-, pressure-, and strain-rate sensitivity, and the effective plastic strain rate is written as

(1) $$\dot{\bar{\varepsilon }}={\dot{\varepsilon }}_{0}\exp\left[-\left(\frac{A\left(s-\alpha \sigma_{c}\right)}{\theta }\right)\left(1-{\left(\frac{\sigma_{\mathrm{eq}}}{s-\alpha \sigma_{c}}\right)}^{m}\right)\right],$$

where $\sigma_{\mathrm{eq}}$ is the equivalent stress, θ is the absolute temperature, and the material constants *m*, $\dot{\varepsilon_{0}}$, and *A* are the rate-dependent sensitivity parameters. The magnitude $\sigma_{c}=tr\left(\sigma \right)$ is the trace of Cauchy stress, and α is the pressure sensitivity constant.

The rate of plastic straining is controlled by the athermal resistance *s* [31] with an initial value $s_{0}$ calculated by

(2) $$s_{0}=s_{1}/1.8\;\mathrm{with}\;s_{1}=\sqrt{3}\frac{8.5^{-1/m}G}{\left(1-\nu \right)},$$

where ν is the Poisson’s ratio and *G* is the shear modulus of the material.

According to the USCP model [28], the extended equation of the athermal resistance evolution $\dot{s}$ was proposed in a more generalized form:

(3) $$\dot{s}=H_{1}\left(\bar{\varepsilon }\right)\left(1-\frac{s}{s_{1}}\right)\dot{\bar{\varepsilon }}+H_{2}\left(\bar{\varepsilon }\right)\left(1-\frac{s}{s_{2}}\right)\dot{\bar{\varepsilon }}+H_{3}\left(\bar{\varepsilon }\right)\left(1-\frac{s}{s_{3}}\right)\dot{\bar{\varepsilon }},$$

where $s_{1}$, $s_{2}$, and $s_{3}$ are athermal strengths that correspond to the preferred state at different stages [28,30]. $s_{1}$ and $s_{2}$ are the athermal strengths related to the peak yield and lower yield. Athermal strength $s_{3}$ is the preferred state of the crystalline phase, and it may depend on temperature, strain rate, crystallinity degree, and humidity. To characterize the transition slopes between different stages, three hardening (or softening) parameters, $h_{1}$, $h_{2}$, $h_{3}$, and the smoothing factor *f* are involved for the calculation of $s_{1}$, $s_{2}$, and $s_{3}$. $H_{1}$, $H_{2}$, and $H_{3}$ are Heaviside-like functions given by:

(4) $$\begin{array}{cc} \; & H_{1}\left(\bar{\varepsilon }\right)=-h_{1}\left(\tanh\left(\frac{\bar{\varepsilon }-{\bar{\varepsilon }}_{p}}{f{\bar{\varepsilon }}_{p}}\right)-1\right),\\ \; & H_{2}\left(\bar{\varepsilon }\right)=h_{2}\left(-\tanh\left(\frac{\bar{\varepsilon }-{\bar{\varepsilon }}_{p}}{f{\bar{\varepsilon }}_{p}}\right)\tanh\left(\frac{\bar{\varepsilon }-{\bar{\varepsilon }}_{c}}{f{\bar{\varepsilon }}_{c}}\right)+1\right),\\ \; & H_{3}\left(\bar{\varepsilon }\right)=h_{3}\left(\tanh\left(\frac{\bar{\varepsilon }-{\bar{\varepsilon }}_{c}}{f{\bar{\varepsilon }}_{c}}\right)+1\right), \end{array}$$

where ${\bar{\varepsilon }}_{p}$ is the plastic strains at the peak yield point and ${\bar{\varepsilon }}_{c}$ is the low yield point just prior to the yielding of crystalline phases taking place.

In summary, the USCP model can describe the complex nonlinear mechanical behavior of polymers. The calculation of *s*, the athermal shear resistance, requires 16 parameters: *G*, ν, ${\dot{\varepsilon }}_{0}$, *m*, *A*, θ, ${\bar{\varepsilon }}_{c}$, ${\bar{\varepsilon }}_{p}$, $h_{1}$, $h_{2}$, $h_{3}$, *f*, $s_{0}$, $s_{1}$, $s_{2}$, and $s_{3}$.

### 2.2. The USCP-Lite Model

As shown in Equation (1), the rate-sensitivity parameters ${\dot{\varepsilon }}_{0}$, *A*, and *m* are integral to the fundamental framework of the USCP model, governing the evolution of the athermal shear stress “*s*”. The identification of these parameters requires the peak stress $\sigma_{p}$, Young’s modulus *E*, and calculated athermal resistance $s_{1}$ corresponding to $\sigma_{p}$ [28,31], which can be directly obtained from stress–strain curves at two different strain rates. However, when the study of strain rate effects is outside the scope of interest, such as in cases where only quasi-static loading conditions are relevant, as often occurs during material characterization under testing standards [32] for many engineering applications at room temperature, the full predictive capabilities of the USCP model may not be necessary or practical.

To address this, this section introduces an alternative version of the USCP model, reformulated to render the polymer response rate-independent. This adaptation simplifies the model for scenarios where strain rate effects are not a primary concern, allowing for a more focused analysis of other material properties under quasi-static conditions.

The initial approach for identifying ${\dot{\varepsilon }}_{0}$ and *A* involves analyzing the relationship between $\ln\dot{\varepsilon }$ and ${\left(\sigma_{p}/s_{1}\right)}^{m}$, where $\dot{\varepsilon }$ is the applied strain rate during the loading test. To initiate the process of identification, Equation (1) can be simplified and rewritten in the following form:

(5) $$\ln\dot{\varepsilon }=B+C{\left(\frac{\sigma_{\mathrm{eq}}}{s-\alpha \sigma_{c}}\right)}^{m},$$

where the intercept *B* and slope *C* are given by:

(6) $$B=\ln{\dot{\varepsilon }}_{0}-\frac{A}{\theta }\left(s-\alpha \sigma_{c}\right),\;C=\frac{A}{\theta }\left(s-\alpha \sigma_{c}\right).$$

When the stress reaches the peak stress, the equations above are specially characterized as $\sigma_{\mathrm{eq}}=\sigma_{p}$ and $s=s_{1}$, where $\sigma_{p}$ is the peak stress. The parameter α accounts for the material’s pressure sensitivity. Our other work includes the characterization of the typical mechanical features of polymers and is currently under review. In this study, pressure sensitivity is not considered, and therefore α=0.

Using the values of *B* and *C*, ${\dot{\varepsilon }}_{0}$ and *A* can be calculated. According to Poulain et al. [31], the parameter *m* corresponds to the values of ${\dot{\varepsilon }}_{0}$ and *A* that best capture the strain-rate sensitivity of the material. Therefore, in this study, the selection of *m* is determined based on the optimal values of ${\dot{\varepsilon }}_{0}$ and *A* that provide the most accurate representation for all the polymer blends simultaneously but with negligible impact on the stress–strain curve when the applied strain rate is changed.

**Figure 2:**
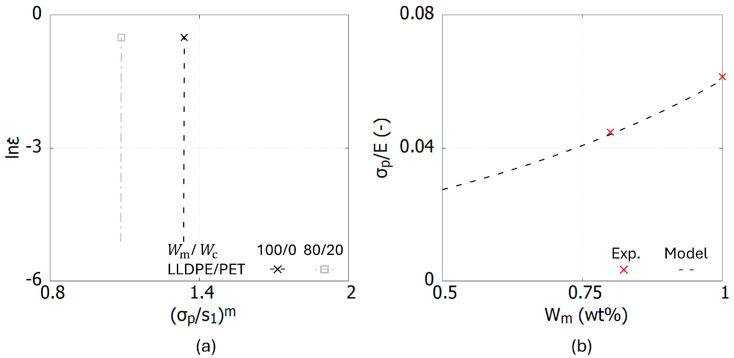
Curves for identifying parameters ${\dot{\varepsilon }}_{0}$, *A*, *m* for LLDPE/PET blends. (**a**) The relationship between $\ln\dot{\varepsilon }$ and ${\left(\sigma_{p}/s_{1}\right)}^{m}$. (**b**) The relationship between $\sigma_{p}/E$ and $W_{m}$.

The experimental data of LLDPE/PET blends [7] are used as example in this section. Figure 2a illustrates the relationship between $\ln\dot{\varepsilon }$ and ${\left(\sigma_{p}/s_{1}\right)}^{m}$ for LLDPE/PET blends with two weight fractions: 100/0 and 80/20. When the rate-sensitivity behavior is not considered, the linear functions are nearly perpendicular to the x-axis. It is important to note that if the curve is perfectly perpendicular to the x-axis, the value of *C* approaches infinity, rendering the triplet (${\dot{\varepsilon }}_{0}$, *A*, *m*) indeterminate. To address this issue, a small offset is introduced to each $\ln\dot{\varepsilon }$ and ${\left(\sigma_{p}/s_{1}\right)}^{m}$ function by setting a sufficiently high value for *C*. In this case, a value of C=2090 was selected. The intercept value *B* varies depending on the type of blend and its weight fraction. To determine *B*, an additional fixed point must be identified and it is explained as follows.

Based on Equations (2) and (5) and the relationship between the elastic modulus and shear modulus G=E/(2(1+ν)), the stress ratio $\sigma_{p}/s_{1}$ is simplified to $\sigma_{p}/kE$, where *k* is a fixed parameter defined as $\sqrt{3}\cdot 8.5^{-1/m}/\left(2(1-\nu )(1+\nu )\right)$. Experimental results in [7] demonstrate that an increase in PET content leads to higher crystallinity in the three polymer blends, resulting in an increase in the Young’s modulus *E*. In other words, the weight fraction of the PET directly influences the stiffness of the polymer blend. The experimental stress ratios, $\sigma_{p}/E$, for the two sampled examined weight fractions are plotted in Figure 2b.

To establish a closure relationship, this work proposes an exponential form expressed as follows:

(7) $$\frac{\sigma_{p}}{E}=ae^{bW_{m}},$$

where *a* and *b* are fixed parameters that can be determined from the Young’s modulus *E* and peak stress $\sigma_{p}$ measurements at two different composition ratios. Here, $W_{m}$ represents the weight fraction of the polymer matrix phase, which contains the minority polymer (hereafter referred to as the “coexisting” phase, $W_{c}$) satisfying the relationship $W_{m}$ + $W_{c}=1$. This formulation allows for a clear distinction between the matrix and contaminant phases, facilitating the analysis of their respective contributions to the material’s mechanical behavior. It is worth noting that both *E* and $\sigma_{p}$ can be easily obtained directly from the stress–strain curve, and simplifying the identification process as these data can be obtained by a regular tensile test under a moderate level of deformation.

Once a and b are identified, Equation (7) is applied to calculate $\sigma_{p}/E$ for various composition ratios of the polymer blends, which are then used to determine *B*. With *C* held constant, ${\dot{\varepsilon }}_{0}$ and *A* can be straightforwardly derived.

## 3. USCP-L Model Application for Contaminated Blends

The USCP-L model is applied to characterize three polymer blends derived from recycling multilayer plastic packaging: (i) LLDPE/PET, (ii) LLDPE/PA6, and (iii) LDPE/PS. For each polymer blend, two weight fractions ($W_{m}/W_{c}=100/0$ and 80/20) are considered. Parameter identification (PI) is carried out by calibrating the USCP-L model directly using stress–strain curves obtained from experimental data available in the literature [7].

This section first outlines the identification results for the rate-sensitivity parameters (${\dot{\varepsilon }}_{0}$, *A*, *m*). The identified set of material constants will be implemented in an in-house finite element (FE) framework for the USCP-L model, utilizing a single-element (SE) configuration. This setup enables rapid evaluation of the stress–strain response in 3D. A summary of the simulation results from this FE model is presented for the three polymer blends studied at the two weight fractions mentioned.

### 3.1. Determination of the Material Constants (${\dot{\varepsilon }}_{0}$, A, m)

As discussed in Section 2.2, the USCP-L model provides a methodology for identifying the rate-sensitivity parameters ${\dot{\varepsilon }}_{0}$, *A*, and *m* by analyzing the relationship between $\sigma_{p}/E$ and the weight fraction of polymer blends. It is important to note that while this set of constants being identified is typically responsible for strain-rate dependency, the USCP-L identification procedure renders the mechanical response rate-independent. This simplification allows the analysis to focus exclusively on the influence of the blend composition ratio, providing clearer insights into how weight fraction affects the mechanical behavior of the blend.

**Figure 3:**
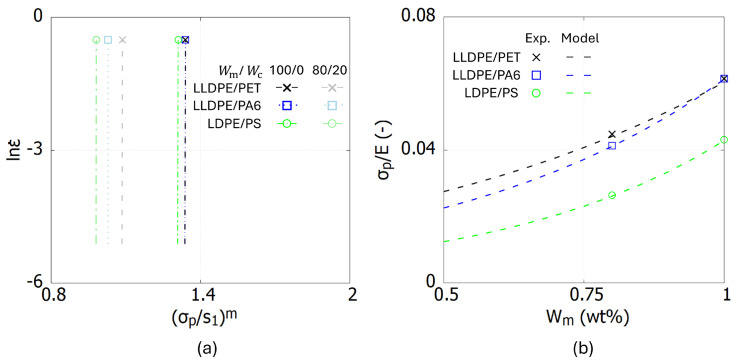
Curves for identifying parameters ${\dot{\varepsilon }}_{0}$, *A*, *m* for three polymer blends. (**a**) The relationship between $\ln\dot{\varepsilon }$ and ${\left(\sigma_{p}/s_{1}\right)}^{m}$. (**b**) The relationship between $\sigma_{p}/E$ and $W_{m}$.

Figure 3a illustrates the relationship between $\sigma_{p}/s_{1}$ and $\ln\dot{\varepsilon }$. The curves are observed to be parallel, and a constant value of C=2090 is assigned to all curves. The intercept *B*, however, varies depending on the polymer blend and its weight fraction. Equation (7) is utilized to characterize $\sigma_{p}/E$ and determine *B* for different composition ratios of polymer blends. The parameters in Equation (7) are identified using *E* and $\sigma_{p}$, both directly extracted from the stress–strain curves corresponding to two weight fractions, $W_{m}/W_{c}=100/0$ and 80/20. The values of *a* and *b* for each blend are listed in Table 1.

Figure 3b compares the experimental data with the predictions of Equation (7) after calibration. The results demonstrate that Equation (7) effectively captures the relationship between $\sigma_{p}/E$ and the weight fraction of the polymer blends. This approach enables the identification of ${\dot{\varepsilon }}_{0}$, *A*, and *m* while maintaining focus on composition-dependent effects.

### 3.2. Calibration of the USCP-L

With the parameters listed in Table 1, the material parameters ${\dot{\varepsilon }}_{0}$ and *A* are calibrated. Then, the calibration of the remaining parameters in the USCP-L model follows the standard PI process [28,31] as follows: (i) the Python script automatically detects the peak yield stress and saturated stress and then with the combination of Equation (2) computes $s_{0}$, $s_{1}$, and $s_{2}$; (ii) hardening and softening parameters $h_{1}$ and $h_{2}$ are calculated considering the transition process of stress increase and drop. (iii) the SE test was employed to determine $s_{3}$, $h_{3}$, and ${\bar{\varepsilon }}_{c}$ [33]. For each weight fraction, the USCP-L model requires only a single stress–strain curve for effective calibration. In this work, three polymer blends with two different weight fractions were considered, and a single parameter set was calibrated for each composition. The calibrated parameters for the USCP model, along with the corresponding thermal properties of the investigated polymers, are provided in Table 2.

### 3.3. USCP-L Finite Element Verification

The USCP-L model and parameter sets presented in Section 3.2 are used to check the mechanical behavior of three polymer blends with mixing ratios of $W_{m}/W_{c}=100/0$ and 80/20.

**Figure 4:**
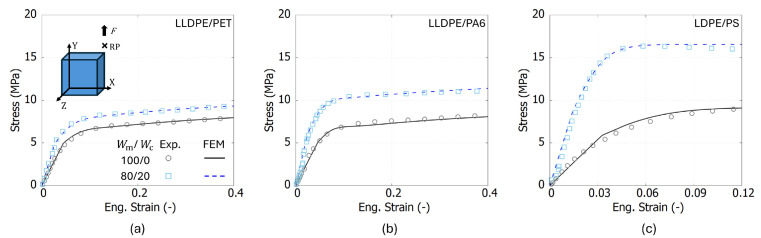
Comparison between the experimental and FEM results of (**a**) LLDPE/PET, (**b**) LLDPE/PA6, and (**c**) LDPE/PS at two mix ratios: $W_{m}/W_{c}=100/0$ and 80/20.

As shown in the inset of Figure 4, an SE test was implemented to iteratively calibrate the model [33]. The simulation employed C3D8R elements, which are eight-node trilinear displacement elements with reduced integration and enhanced hourglass control. To enforce boundary conditions (BCs), one-eighth cube geometrical symmetry was applied. Displacement control was imposed on the top surface by linking it to a reference point (RP), while structural symmetry was maintained by constraining the corresponding degrees of freedom on the left, rear, and bottom surfaces.

Figure 4 presents the FEM results from the USCP-L model, which show good agreement with experimental data. The relative error of each point in the stress–strain curve is below 12%. The model correctly captures the mechanical features such as the yielding and hardening response at the two studied weight fractions. These simulation results will be used as a base for the training of the NN-based constitutive model in the next section.

## 4. NN-Based Constitutive Model and NN Training Process

The internal variable of athermal shear resistance in the USCP-L model, *s*, serves as a fundamental evaluation metric that encapsulates the strain hardening and softening phenomena. According to the variability in the complexity of stress–strain curve shapes, it becomes essential to reformulate the evolution of *s* (refer to Equation (3)) to ensure its applicability under different weight fraction for polymer blends. While adjusting parameter sets calibrated for specific loading conditions can achieve this adaptation, it demands substantial PI efforts.

More critically, this approach does not facilitate a seamless transition when the variability of the weight ratio of the secondary polymer phase is the key parameter in the blend. Following traditional calibration procedures that use experimental data, indeed different sets of material constants are obtained that have to be explicitly input into structural models. In other words, assuming a practical scenario, the cost of a product (e.g., plastic frames for furniture, appliances, vehicle components, etc.) may be reduced by altering the proportions of a plastic alloy, for instance shifting from a 95/5 ratio to 75/25. However, verifying whether the existing design remains valid for this new alloy ratio requires a thorough experimental characterization of the 75/25 mixture. The calibrated parameters are then reintroduced into the finite element model to re-evaluate the structure’s mechanical behavior and determine the acceptability of the new blend. In the absence of a constitutive model capable of predicting the stress–strain curve for this adjusted ratio, the process of specimen fabrication, mechanical testing, and model calibration must be repeated. Such a process is time-consuming, resource-intensive, and can hinder competitiveness, thereby complicating the adoption of more sustainable materials without sacrificing quality.

To address this limitation, a practical and efficient NN-based constitutive model is introduced. The structure of the NN-based model is outlined in the flowchart shown in Figure 1, which complies with the USCP-L model framework. This NN is tailored specifically to track the evolution of the internal variable “*s*” across varying ratios of polymer proportions in the blend. Additionally, to ensure precise predictions of the mechanical behavior of the blend at different mixing ratios, a systematic calibration of the relationship between Young’s modulus and the weight fraction is essential.

This section begins by describing how the dataset is prepared. It then introduces a phenomenological expression designed to capture the relationship between the Young’s modulus and the composition of polymer blends. This is followed by a detailed explanation of the selected NN structure and how the training was conducted. As a continuation of this section, the subsequent Section 5 will show how the NN-based constitutive model implemented as an FE user-defined material performs, henceforth referred to as “FEM-NN”. This model is directly compared with the traditional FE constitutive model presented in Section 3.3, which is denoted as “FEM”.

### 4.1. Dataset Preparation

The FEM results generated from the SE test models were collected and used as datasets for training and verifying the NN-based constitutive model. The input dataset of each polymer blend consists of two input parameters: ε and $W_{m}$. ε represent the current strain during the loading process, while $W_{m}$ denotes the weight ratio of the main composition in the polymer blend. To obtain the strain value as an input for the NN, the equivalent strain $\varepsilon =\sqrt{\left(2/3\right)\mathrm{trace}\left(\varepsilon :\varepsilon^{T}\right)}$ is used in this paper.

For each polymer blend, the training dataset includes two weight fractions (100/0 and 80/20), with 400 data points from the stress–strain curve collected per blend, totaling 800 datasets for training. The collected data are then normalized and scaled using the Min-Max scaling method, ensuring that all values are within the range of [0,1]. Subsequently, the dataset is split into training (75%) and validation (25%) sets.

### 4.2. Calibration of E

With the addition of a coexisting polymer phase, the morphology and the mechanical properties of material are influenced. Experimental data indicate that if a polymer blend has a composition in which one polymer dominates (either by weight fraction or due to its low viscosity), it tends to form a continuous phase (matrix), thereby isolating the other polymer phase into discrete particles. The resulting morphology plays a critical role in determining the capability of deformation and its ultimate mechanical properties [7,34].

Several studies have investigated the relationship between the overall elastic modulus *E* of polymer blends and the volume fraction of each component (see, for example, [35,36]). In practical manufacturing, however, weight fractions are more commonly used. Therefore, converting weight fractions to volume fractions can be as follows:

(8) $$V_{c}=\frac{W_{c}\rho_{c}}{W_{c}\rho_{c}+W_{m}\rho_{m}},$$

where $V_{c}$ ($V_{m}$) represents the volume fraction of the coexisting polymer (matrix) and $\rho_{c}$ and $\rho_{m}$ are their corresponding densities. Additionally, to estimate how the overall elastic modulus *E* depends on the mixture ratio in the blend, theoretical models are often employed. The most popular and practical to provide the upper and the lower bounds of *E* are those commonly known as the “Rule of Mixtures”, which are expressed as follows:

(9) $$E=V_{m}E_{m}+V_{c}E_{c},\;E=\frac{E_{m}W_{m}\rho_{m}+E_{c}W_{c}\rho_{c}}{W_{c}\rho_{c}+W_{m}\rho_{m}},$$

and

(10) $$\frac{1}{E}=\frac{V_{m}}{E_{m}}+\frac{V_{c}}{E_{c}},\;E=\frac{E_{m}E_{c}\left(W_{c}\rho_{c}+W_{m}\rho_{m}\right)}{E_{c}W_{m}\rho_{m}+E_{m}W_{c}\rho_{c}}.$$

Here, $E_{m}$ and $E_{c}$ denote the Young’s moduli of the pure matrix and coexisting polymers, respectively, while *E* is the overall blend modulus. Equation (9) corresponds to the Voigt model (a parallel arrangement) [37], which provides an upper bound for the modulus. In contrast, Equation (10) represents the Reuss model (an inverse or series arrangement) [38], yielding a harmonic mean of the moduli weighted by the respective volume fractions. For higher concentration and co-continuous morphologies, Davies et al. [39] proposed the following equation to calculate the Young’s modulus:

(11) $$E^{1/5}=V_{m}E_{m}^{1/5}+V_{c}E_{c}^{1/5},\;E^{1/5}=\frac{W_{m}\rho_{m}E_{m}^{1/5}+W_{c}\rho_{c}E_{c}^{1/5}}{W_{m}\rho_{m}+W_{c}\rho_{c}}$$

The experimental data utilized in this study have been extracted from the work of Demets et al. [7]. Figures 8a,b and 9c of that research [7] present SEM micrographs of LLDPE/PA6, LLDPE/PET, and LDPE/PS blends at a weight ratio of $W_{m}:W_{c}=80/20$. These micrographs clearly present PET and PA6 droplets dispersed within the LLDPE/PET and LLDPE/PA6 blends. In contrast, the LDPE/PS blend exhibits a partially co-continuous structure, yet the two distinct phases of LDPE and PS remain identifiable. Consequently, in this study, these polymer blends are treated as composite systems consisting of two distinct phases within the weight fraction range of $W_{m}/W_{c}=\left[80/20,100/0\right]$. To predict the relationship between *E* and the mix ratios of each composition, the Halpin–Tsai equations [40,41] are applied to the three polymer blends in this study. Originally formulated for semi-crystalline polymers based on a two-phase composite system assumption, the Halpin–Tsai equations have also been applied by Tai et al. [42] to LDPE- and HDPE-based blends. Their results demonstrated good agreement between the predicted and measured values. These equations incorporate the contributions of each composition are given as follows:

(12) $$E=E_{m}\left(\frac{1+\xi \eta V_{c}}{1-\eta V_{c}}\right),\;E=E_{m}\left(\frac{1+\xi \eta \frac{W_{c}\rho_{c}}{W_{c}\rho_{c}+W_{m}\rho_{m}}}{1-\eta \frac{W_{c}\rho_{c}}{W_{c}\rho_{c}+W_{m}\rho_{m}}}\right)\;\mathrm{with}\;\eta =\frac{E_{c}/E_{m}-1}{E_{c}/E_{m}-\xi }$$

where ξ is the geometry parameter. In this study, weight fractions are utilized to express different composition ratios in polymer blends.

**Figure 5:**
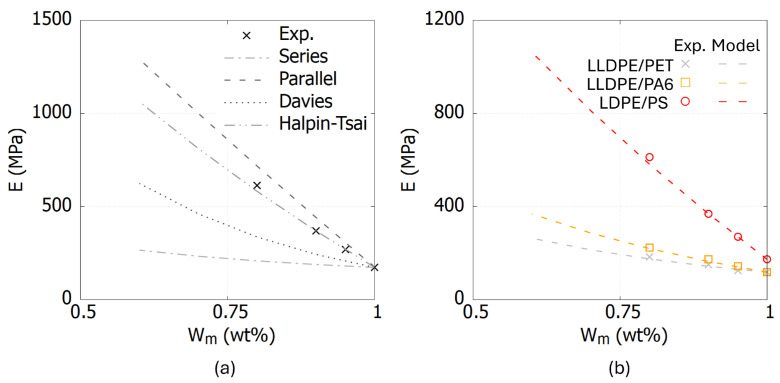
The relationship between *E* and the amount of main composition of polymer blends $W_{m}$. (**a**) The comparison of the *E* values for LDPE/PS blends between experimental results and common model. (**b**) The comparison between *E* from experimental data and the Halpin–Tsai equations.

Figure 5a presents a comparison of the *E* values for LDPE/PS blends between experimental results and predictions from the Voigt, Reuss, Davies, and Halpin–Tsai models. The comparison demonstrates that the Halpin-Tsai equations exhibit good agreement with the experimentally obtained *E* values. Furthermore, the Halpin–Tsai models also applied to LLDPE/PET and LLDPE/PA6 blends. ξ depends on the aspect ratio and geometry of the coexisting polymer phases and the value is positive. The value of ξ is shown in Table 3 and the comparison between *E* from experimental data and the Halpin–Tsai equations is shown in Figure 5b.

### 4.3. NN Structure

A fully connected NN was used in this paper to train the internal athermal shear stress function *s*. For three polymer blends, the mean squared error (MSE = $\frac{1}{n}\sum_{i=1}^{n}{\left(y_{i}-{\hat{y}}_{i}\right)}^{2}$) was selected as the loss function, with the tanh function chosen as the activation function. The learning rate was set to 0.0001, and the Adam optimizer, an adaptive learning rate optimization algorithm, was used. These NN structures were implemented using PyTorch 2.0.1 with Python 3.10. The value of *s* was replaced by the NN, and the equation is expressed as follows:

(13) $$z^{\left(l\right)}=W^{\left(l\right)}a^{\left(l-1\right)}+b^{\left(l\right)}$$

(14) $$a^{\left(l\right)}=tanh\left(z^{\left(l\right)}\right)$$

where $z^{\left(l\right)}$ represents the linear output (pre-activation) of the *l*-th layer, $W^{\left(l\right)}$ is the weight matrix, $a^{\left(l-1\right)}$ is the activated output from the previous layer, $b^{\left(l\right)}$ is the bias vector, and $a^{\left(l\right)}$ denotes the activated output using the activation function tanh in the *l*-th layer.

As introduced in Section 4.1, each polymer blend dataset only contains 800 sets of data. To improve the efficiency of the trainnig process and the generalization of the NN, *k*-fold cross-validation is employed during the training process for all polymer blends.

**Figure 6:**
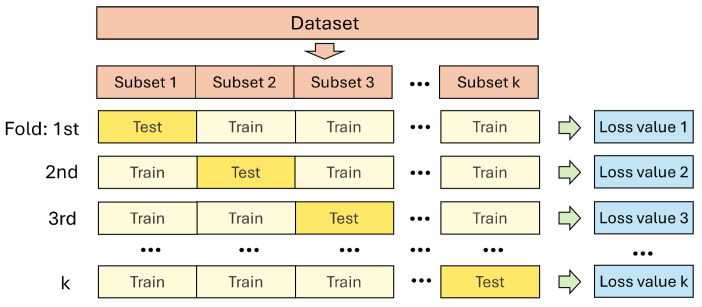
Schematic diagram of *k*-fold cross-validation, where the dataset is randomly divided into *k* subsets during the training process. Each subset is used as a test set once, while the remaining k−1 subsets are used for training. This process is repeated *k* times, with two loss values during training and validation process being output after each training iteration.

The *k*-fold cross-validation is a fundamental tool used to evaluate the performance of machine learning models, particularly when dealing with small datasets. Figure 6 illustrates the fundamental concept of *k*-fold cross-validation. The dataset is randomly divided into *k* subsets. During the training process, the NN model is trained *k* times, each time using k−1 folds subsets for training and the remaining fold for testing. This approach maximizes data utilization and reduces variance in performance estimation.

Different NN structures were tested for the three polymer blends, and based on the learning rate and evaluation results, a five-layer NN structure (2-6-9-12-6-1) was adopted for LLDPE/PET and LDPE/PS, while a five-layer NN structure (2-4-6-8-4-1) was used for LLDPE/PA6.

### 4.4. NN Training Process

**Figure 7:**
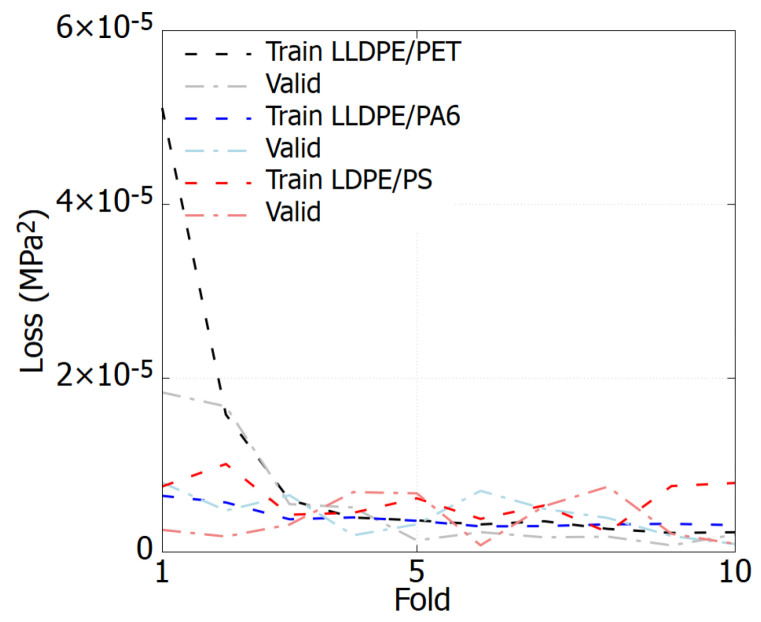
The learning curve for three polymer blends, each represented by two curves corresponding to the training and validation processes. During training, the loss value decreases rapidly at first and then gradually stabilizes.

For all cases, the training process involves 300 epochs for each iteration within a *k*-fold cross-validation, where *k* is set to 10. Figure 7 displays the learning curves for each polymer blend throughout the training sessions. The results show the loss values after each fold, illustrating that the loss tends to stabilize and decreases to $10^{-5}$ by the end of the training. This pattern indicates strong convergence for the model across all polymer blends.

## 5. Validation and Prediction of the FEM-NN Results

After training, three pre-trained NNs were integrated into the FEM through a user-defined material subroutine, referred to as the FEM-NN model, to accurately capture the specific mechanical properties of the material. The same SE test was conducted using the FEM-NN model to validate its performance.

In this section, a comparison between the FEM-NN and FEM results is presented to evaluate the accuracy of the FEM-NN model. Furthermore, to assess its generalization capability, the FEM-NN model was applied to predict the mechanical behavior of the polymer blends with unseen weight fractions, demonstrating its robustness and adaptability to other weight fractions.

### 5.1. Validating Results

**Figure 8:**
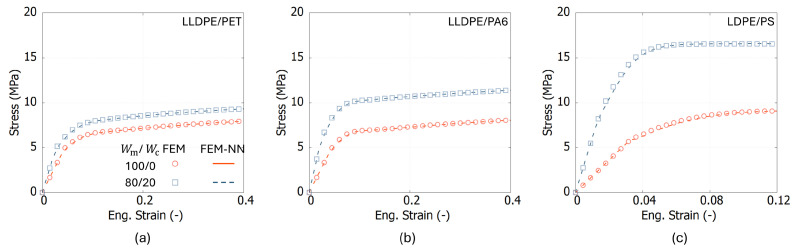
The comparison results between FEM and FEM-NN for (**a**) LLDPE/PET, (**b**) LLDPE/PA6, and (**c**) LDPE/PS at two mix ratios: $W_{m}/W_{c}=100/0$ and 80/20.

Figure 8 illustrates the stress–strain curves obtained from both FEM and FEM-NN simulations for three polymer blends at two weight fractions, $W_{m}/W_{c}$ = 100/0 and 80/20. It can be observed that the FEM-NN results exhibit good agreement with the FEM results, suggesting that the FEM-NN model captures the mechanical behavior as good as the traditional FEM approach. The relative error of each point in the stress–strain curve between the FEM and FEM-NN results is below 4%.

### 5.2. Predicting Results

To evaluate whether the FEM-NN model can adapt to other mixing ratios beyond $W_{m}/W_{c}$ = [100/0, 80/20], experimental results from three polymer blends with $W_{m}/W_{c}$ = 95/5 and 90/10 are utilized to assess the generalization capability of the FEM-NN model. This evaluation aims to validate the model’s ability to accurately predict mechanical behavior for unseen weight fractions, further demonstrating its robustness and applicability to a wider range of material compositions.

**Figure 9:**
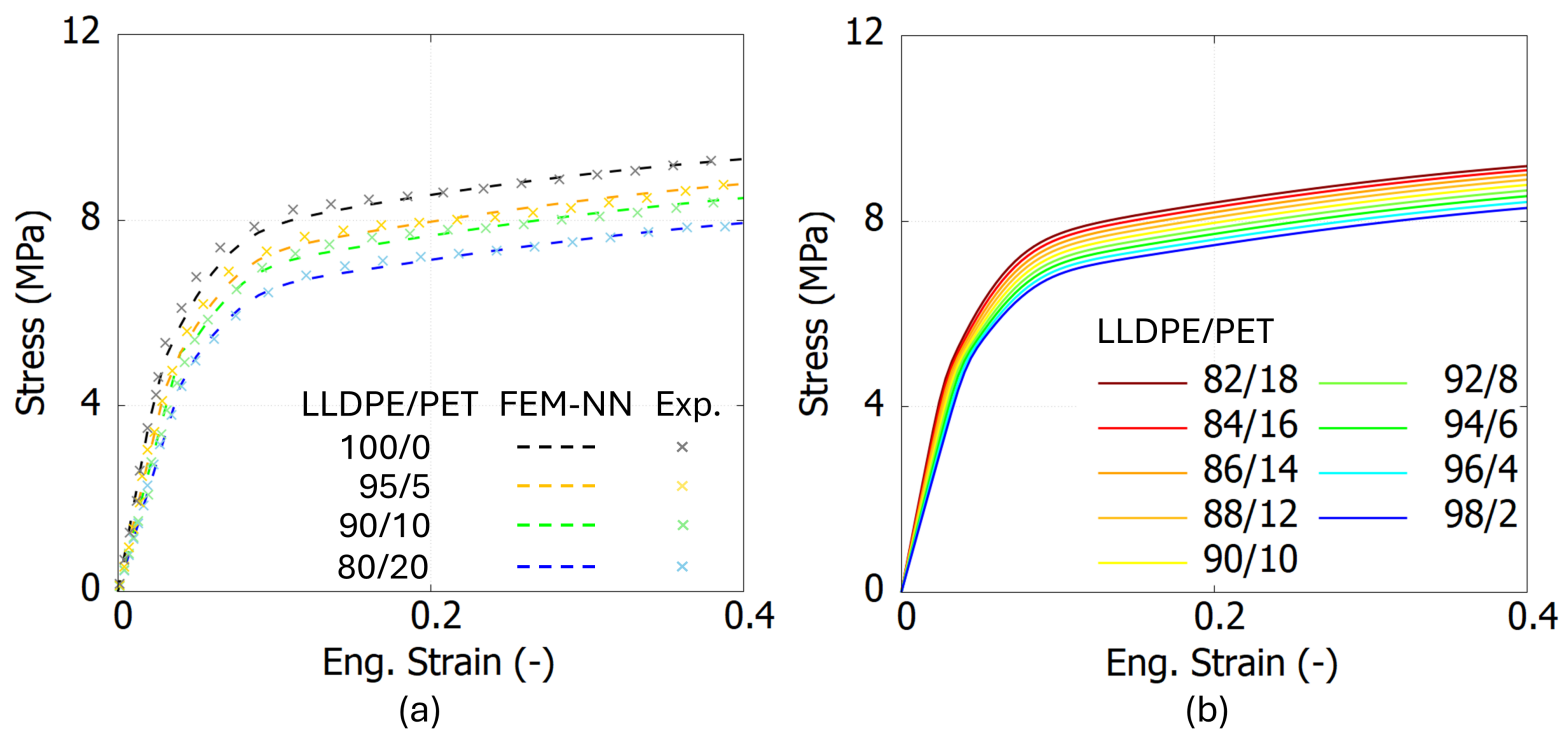
The experimental and prediction results from FEM-NN model for LLDPE/PET blends. (**a**) The comparison between FEM-NN and experimental results. (**b**) The prediction results from FEM-NN between $W_{m}/W_{c}$ = [98/2, 82/18].

**Figure 10:**
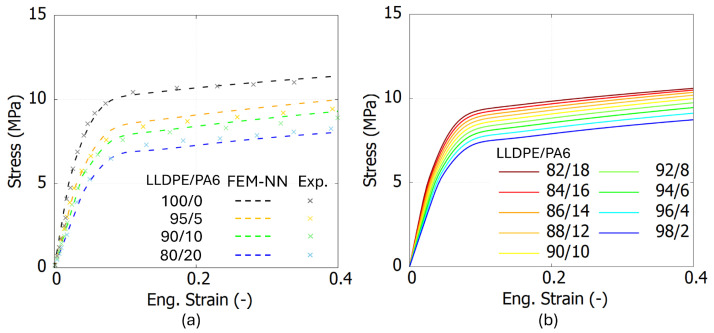
The experimental and prediction results from FEM-NN model for LLDPE/PA6 blends. (**a**) The comparison between FEM-NN and experimental results. (**b**) The prediction results from FEM-NN between $W_{m}/W_{c}$ = [98/2, 82/18].

**Figure 11:**
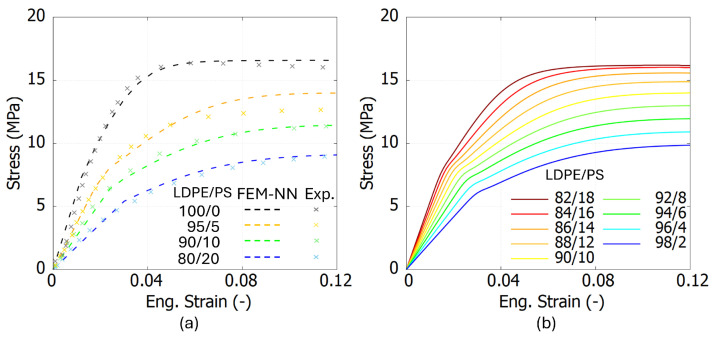
The experimental and prediction results from FEM-NN model for LDPE/PS blends. (**a**) The comparison between FEM-NN and experimental results. (**b**) The prediction results from FEM-NN between $W_{m}/W_{c}$ = [98/2, 82/18].

To evaluate the accuracy of the FEM-NN model, Figure 9a, Figure 10a and Figure 11a present the comparison results between the experiments and the FEM-NN model. These three figures show that the prediction results are consistent with the experimental data in both the elastic and inelastic stages. However, as the strain increases, the predictions from the FEM-NN model are slightly higher than the experimental results for a mix ratio of $W_{m}/W_{c}$ = 90/10, especially for LDPE/PS blends. Nevertheless, the relative error remains below 15%. This phenomenon may be attributed to the morphological changes induced by the increasing addition of PS, as evidenced by SEM images [7]. A significant increase in the crystallinity of the LDPE matrix and *E* is observed when the contamination level reaches 20 wt%. In the case of PA6- and PS-contaminated blends, the LDPE/PS blend transitions into a partially co-continuous structure, further influencing its mechanical properties.

To evaluate whether the FEM-NN model can achieve a smooth transition of predicted stress–strain curves within a specific range, Figure 9b, Figure 10b and Figure 11b present the stress–strain curves predicted by the FEM-NN model for intermediate weight fractions between $W_{m}/W_{c}$ = [98/2, 82/18]. The results demonstrate that the FEM-NN model successfully generates smooth and continuous transitions in the stress–strain behavior, indicating its capability to interpolate mechanical properties across varying compositions.

In summary, the FEM-NN model demonstrates the capability to replace traditional FEM models for SE test simulations while offering enhanced adaptability to various loading conditions. This underscores the potential of the FEM-NN constitutive model as a versatile and cost-effective framework for simulating complex material behaviors, requiring only a minimal and affordable amount of experimental testing and characterization.

## 6. Conclusions

In this study, we have introduced a cost-effective hybrid modeling methodology that integrates a physics-based constitutive model (the Unified Semi-Crystalline Polymer model, USCP) with neural networks (NNs) and finite element modeling (FEM). This integrated approach effectively predicts mechanical stress–strain responses of polymer blends comprising a primary component (LLDPE or LDPE) and secondary polymers (PA6, PET, or PS) at varying weight fractions.

The principal advancements and contributions include:

1. Development of the USCP-Lite (USCP-L) model: We have enhanced and generalized the original USCP model to the USCP-L variant, tailored specifically for polymers that exhibit negligible or no strain-rate sensitivity. This streamlined model successfully characterizes the mechanical behaviors of different polymer blends at multiple weight fractions.
2. Incorporation of weight fraction effects via NN: A neural network-based constitutive approach has been introduced, enabling efficient integration of polymer blend weight fractions directly into predictive modeling. This NN-enhanced constitutive model has been applied to various recycled polymer blends commonly used in industry, where the primary polymer includes up to 20% of a secondary component.
3. Integration strategy with FEM: We have implemented a direct integration procedure that allows the NN-based constitutive model (FEM-NN) to function seamlessly within conventional FEM frameworks as a user-defined material, enabling structural-level simulations with complex geometries.
4. Model validation: The accuracy and versatility of the FEM-NN model have been validated against experimental data for three common polymer blends, LLDPE/PET, LLDPE/PA6, and LDPE/PS, across weight ratios ranging from pure polymers (100/0) to blends (80/20), confirming strong predictive capabilities.

The outcomes of this research hold substantial industrial applicability, especially within sectors increasingly utilizing recycled polymer blends. For example, consider the packaging industry, which frequently incorporates blends such as LLDPE/PET, LLDPE/PA6, and LDPE/PS in sustainable product designs. Our FEM-NN model provides engineers with a versatile predictive tool to virtually evaluate mechanical performance across relevant blend compositions minimizing the amount of experimental trials. This capability significantly enhances the efficiency of material selection processes, streamlines product development, and reduces both time and cost associated with traditional trial-and-error testing.

Despite its strengths, the FEM-NN model currently does not explicitly account for microstructural changes in polymer blends, resulting in minor discrepancies between predictions and experimental results for certain compositions. To address this, future developments will enhance the neural network dataset by including additional influential parameters such as additive content, crystallinity levels, and detailed morphological characteristics. Moreover, the model will be expanded to accurately represent multi-component polymer blends. Finally, the practical USCP-L model is recommended for scenarios involving rate-independent behavior or when experimental data at different loading rates are unavailable or considered secondary. Conversely, for cases with significant rate-dependent behavior, the original, comprehensive USCP model remains the preferred choice.

## Figures and Tables

**Table 1:** Parameters of Equation (7) for the $E-W_{m}$ relationship of three polymer blends.

Materials	*a*	*b*	*m*
LLDPE/PET	0.0125	1.5754	0.66
LLDPE/PA6	0.0083	1.9984	0.66
LDPE/PS	0.0036	2.4758	0.59

**Table 2:** Set of identified parameters required for the USCP model for LLDPE/PET, LLDPE/PA6, and LDPE/PS blends.

Material Parameter	Unit	Description	LLPDE/PET		LLPDE/PA6		LPDE/PS	
			100/0	80/20	100/0	80/20	100/0	80/20
*E*	MPa	Young’s modulus	113	184	113	250	183	613
υ	-	Poisson’s ratio	0.38		0.38		0.38	
$s_{0}$	MPa	Initial shear strength	2.48	4.04	2.48	5.49	2.74	9.17
$s_{1}$	MPa	Pre-peak strength	4.47	7.28	4.47	9.89	4.93	16.5
$s_{2}$	MPa	First saturation strength	5.2	7.8	5.2	13	5.2	16.8
$s_{3}$	MPa	Second saturation strength	7	9.8	7	15	6.3	17
$h_{1}$	MPa	Pre-peak hardening	125	185	225	365	240	1450
$h_{2}$	MPa	Post-peak softening	25	60	35	35	200	503
$h_{3}$	MPa	Second yield hardening	11	15	11	8	190	1060
${\bar{\varepsilon }}_{p}$	-	Peak plastic strain	0.0705	0.0698	0.0655	0.0879	0.00581	0.01024
${\bar{\varepsilon }}_{c}$	-	Activation plastic strain	0.0715	0.0701	0.0665	0.08822	0.00582	0.01028
*f*	-		0.3		0.3		0.3	
*m*	-	Rate sensitivity	0.66		0.66		0.59	
${\dot{\varepsilon }}_{0}$	$s^{-1}$	Rate sensitivity	$6.96\times 10^{-296}$	$1.83\times 10^{-70}$	$1.19\times 10^{-306}$	$8.9\times 10^{-26}$	$7.6\times 10^{-289}$	$1.82\times 10^{14}$
*A*	K/MPa	Rate sensitivity	$1.38\times 10^{5}$	$8.5\times 10^{4}$	$1.38\times 10^{5}$	$6.26\times 10^{4}$	$1.25\times 10^{5}$	$3.76\times 10^{4}$

**Table 3:** Parameters of the $E-W_{m}$ relationship for three polymer blends.

Materials	$\rho_{m}$ ($\mathrm{kg}\cdot m^{-3}$)	$\rho_{c}$ ($\mathrm{kg}\cdot m^{-3}$)	ξ
LLDPE/PET	925	880	1.4
LLDPE/PA6	925	1130	4.9
LDPE/PS	920	1048	42

## Data Availability

Data are contained within the article.
